# Drought- and soil substrate-induced variations in root nonstructural carbohydrates result from fine root morphological and anatomical traits of *Juglans mandshurica* seedlings

**DOI:** 10.1186/s12870-022-03987-x

**Published:** 2023-02-07

**Authors:** Li Ji, Jun Wang, Yue Liu, Zhimin Lu, Witoon Purahong, Yuchun Yang

**Affiliations:** 1 School of Forestry, Central South University of Forestry and Technology, 410004 Changsha, P.R. China; 2 Jilin Academy of Forestry, 130033 Changchun, P.R. China; 3 UFZ-Helmholtz Centre for Environmental Research, Department of Soil Ecology, Theodor- Lieser-Str. 4, 06120 Halle (Saale), Germany

**Keywords:** Fine root, Nonstructural carbohydrate, *Juglans mandshurica*, Drought, Soil substrates, Functional traits

## Abstract

**Background:**

Nonstructural carbohydrates (NSCs) reflect the carbon supply status and affect the construction and development of plants. Previous studies have focused on the dynamics of NSCs among plant organs, however, few studies have paid attention to the synergistic variations between fine root traits and NSCs under drought based on the perspective of branch order roots. This study aims to explore the responses of fine root traits and NSCs among root orders of *Juglans mandshurica* seedlings under different drought intensities and soil substrates. The 2-year-old *J. mandshurica* potted seedlings were planted in three different soil substrates (humus, loam and sandy-loam soil) and subjected to four drought intensities (CK, mild drought T1, moderate drought T2 and severe drought T3) for 60 days.

**Results:**

The root biomass of seedlings in sandy-loam soil under the same drought intensity was higher than that of seedlings in humus soil. With an increase in drought, the root biomass, average diameter, root tissue density and cortex thickness decreased significantly, and the specific root length, stele diameter and conduit density increased. The root NSC contents in humus soil were higher than those in sandy-loam soil. The fine root soluble sugar content in all soil substrates decreased with increasing drought intensity, while the root starch and total NSC contents varied among the different soil substrates. Compared with transportive roots, the morphological and anatomical traits jointly explained the higher variation in NSC contents of the absorptive roots. The anatomical traits explained the higher variation in the NSC content of first five order roots.

**Conclusion:**

Our results suggest that coordinated adaptation of the root traits and NSCs of Manchurian walnut seedlings exposed to water gradients in different soil substrates.

**Supplementary Information:**

The online version contains supplementary material available at 10.1186/s12870-022-03987-x.

## Background

In recent decades, ongoing high temperatures and frequent drought events worldwide have strikingly affected plant growth and survival [1, 2]. Drought is the prevalent environmental factor limiting forest productivity, and it directly causes the mortality of trees and the failure of seedling germination, and indirectly makes plants more vulnerable to pathogens or insects [2, 3]. Therefore, there is an urgent need to better decipher how plants respond to drought as well as to cultivate plants with greater drought tolerance [4]. Roots are the main organs for substrate and energy exchange at the interface of plants and soil, and they perform crucial tasks in the absorption and transportation of water and nutrients [5, 6]. When exposed to drought, the increased resource allocation for root growth usually facilitates water acquisition. Therefore, the plasticity of root system is pivotal to the plant acclimation and survival in adverse environments [7].

Fine roots (diameter < 2 mm) have a complex hierarchical structure, and mounting evidence has shown a strong linkage of root function with root branch order [8–10]. Based on the perspective of root anatomy, previous studies demonstrated that branch order can serve as an effective approach to distinguish the absorptive roots and transport roots [10, 11]. Root morphological traits characterize the water transport efficiency and absorption capacity of the root system [12], while root anatomical structures affect radial and axial water transportation [13]. Amounting evidence has confirmed that a trade-off exists between root growth and exploration when plants suffer from drought. For instance, Chimungu et al. [14] found that a reduced number of cortical cells improves drought tolerance by reducing the metabolic cost of resource exploration. Burton et al. [15] revealed that wild corn (*Zea* spp.) reduced growth and metabolic costs (carbon consumption for root respiration and nutrients for living tissues) by reducing the proportion of active cortical tissue to the root volume to maintain root growth under drought conditions. In addition, Yamauchi et al. [16] found that the cortex–to–stele ratio, xylem–to–stele ratio and aerenchyma–to–cortex ratio could explain the adaptability of wild Poaceae species to soil water gradients. Previous studies revealed the effects of drought on the root tip or the entire root systems [17, 18]; however, to the best of our knowledge, few studies have been conducted on the differential responses of fine root traits to drought among branch orders [19]. Therefore, unravelling the response of fine roots to changing water availability based on the perspective of root orders is crucial for understanding the resource utilization strategies and performance of plants under future climate change.

Nonstructural carbohydrates (NSCs, mainly comprising soluble sugars and starch) are important substrates of the plant carbon budget under drought conditions [20, 21]. Except for tree trunks, roots have the highest NSC contents [22], and root NSCs are constantly consumed by fine root production, respiration metabolism, and osmotic regulation [23]. Previous studies have proven that root growth and soil exploration consume more than 50% of the photosynthesized carbohydrates [24]. Hartmann et al. [25] found that lethal drought reduced the soluble sugar (SS) and starch (ST) contents of roots, but the subsequent rewatering treatment did not result in a significant change in root NSC contents [26]. Nevertheless, the response of NSC variation based on different branch orders to drought has rarely been reported. More recently, Liu et al. [27] applied ^13^ C isotope labelling technology and found that compared with higher orders, drought promoted the structural growth of lower-order roots by increasing the respiration rate and allocating more photosynthetic carbohydrates, but there is still a lack of more fine-scale differences in the allocation of root NSCs. Yang et al. [28] found that the sensitivity of the root NSC concentration of *Phyllostachys edulis* seedlings to drought supported the plasticity of root architecture to some extent in allocating more carbon to structure low-cost roots. Our previous study demonstrated that the root morphological traits but not chemical traits, explain the variation in NSC contents induced by drought [29, 30]. Given the heterogeneity of morphological and physiological function among branch orders [9], however, fewer works on the modifications of NSCs induced by drought have focused on the changes in root morphology and anatomy. We expect that the root NSCs vary highly under drought conditions and may be related to the synergetic adaptation of root morphological and anatomical traits.

*Juglans mandshurica* is a valuable timber species in Northeast China. Increasing evidence suggests that its root system has a branching order with a primary (nonwoody) structure, and its branching order develops into woody roots through secondary development [9, 31, 32]. *J. mandshurica* plantations are distributed in north–south latitudes in Northeast China, and the inhabiting soil types are roughly divided into three categories, namely, humus soil, loam soil and sandy-loam soil. The soil exhibits highly different chemical and physical conditions, and the mechanical impedance of root growth is one of the most constraints that determine root elongation and proliferation along the soil profiles [33]. The soil mechanical resistance increases with decreasing water content in most soil types [34], and the soil types interacted with water stress may affect the morphology, structure and function of roots [33, 35]. Although previous studies have reported the effects of soil types on the morphology and physiology of fine roots [36–38], there is no clear consensus on the magnitude of this combined effect on fine root NSCs or the potential relationship with root traits. Thus, we examined two-year-old potted Manchurian walnut seedlings under three typical soil substrates with different drought intensities to elucidate the variation in fine root traits and NSCs among branch orders. We hypothesized that: (1) drought synergistically affects the variation in fine root morphological and anatomical traits, and the variation in lower-order roots will be higher than that of higher-order roots; (2) with an increase in water deficit, fine root SS, ST and NSC will decrease, and nutrient-rich humus soil has a higher NSC content; (3) due to lower-order roots with the feature of shorter lifespan and faster turnover rate [10], the root NSCs among different branch orders have differential responses to drought intensities and soil substrates, and the degree of variation in lower-order root NSC is higher; and (4) the fine root morphological and anatomical traits jointly exhibit an association with variation of fine root NSC.

## Results

### Effect of drought and soil substrate on fine root biomass and morphological traits

**Fig. 1:**
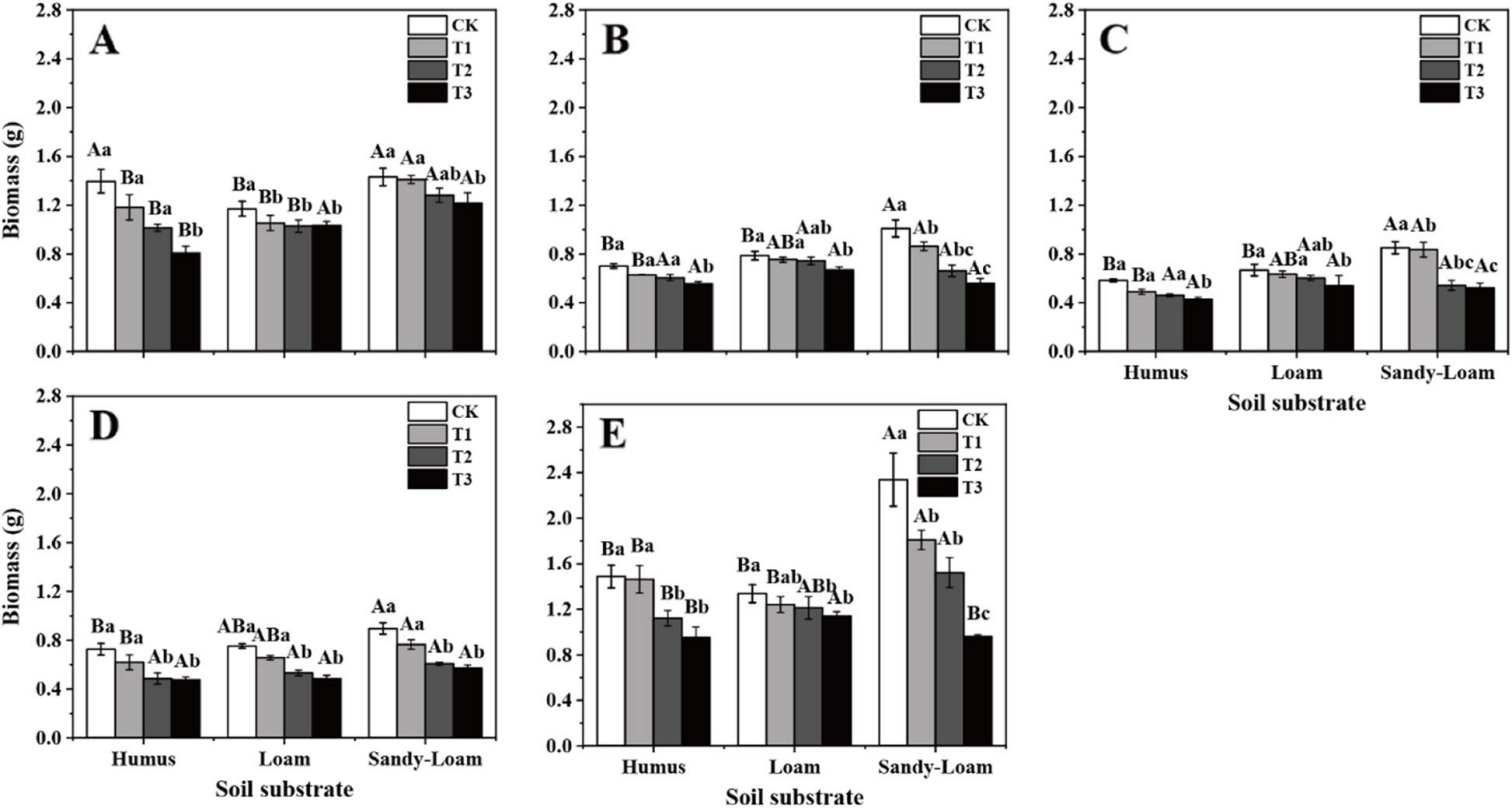
The fine root biomass of *J. mandshurica* seedlings in different drought intensities and soil substrates. Different lowercase letters indicate significant differences among drought intensities (*P* < 0.05). Different uppercase letters indicate significant differences among soil substrates (*P* < 0.05). **A** 1st order root, **B** 2nd order root, **C** 3rd order root, **D** 4th order root, **E** 5th order root. *CK* control, *T1* mild drought, *T2* moderate drought, *T3* severe drought

Fine root biomass was significantly affected by drought and soil substrate among root branch orders (*P* < 0.01, Table S1). Except for first-order roots, the fine root biomass under CK and T1 in sandy-loam soil was higher than that in humus soil (*P* < 0.05, Fig. 1A-E). With increasing drought intensity, the first five order roots biomass decreased progressively. Specifically, except for the first-order root, the coefficient of variation of first-five order roots biomass among different drought intensities in sandy-loam soil was highest: 29.3, 44.4, 47.1, 33.9 and 59.1% in the first five order roots, respectively (Fig. 1A ~ 1E).

**Fig. 2:**
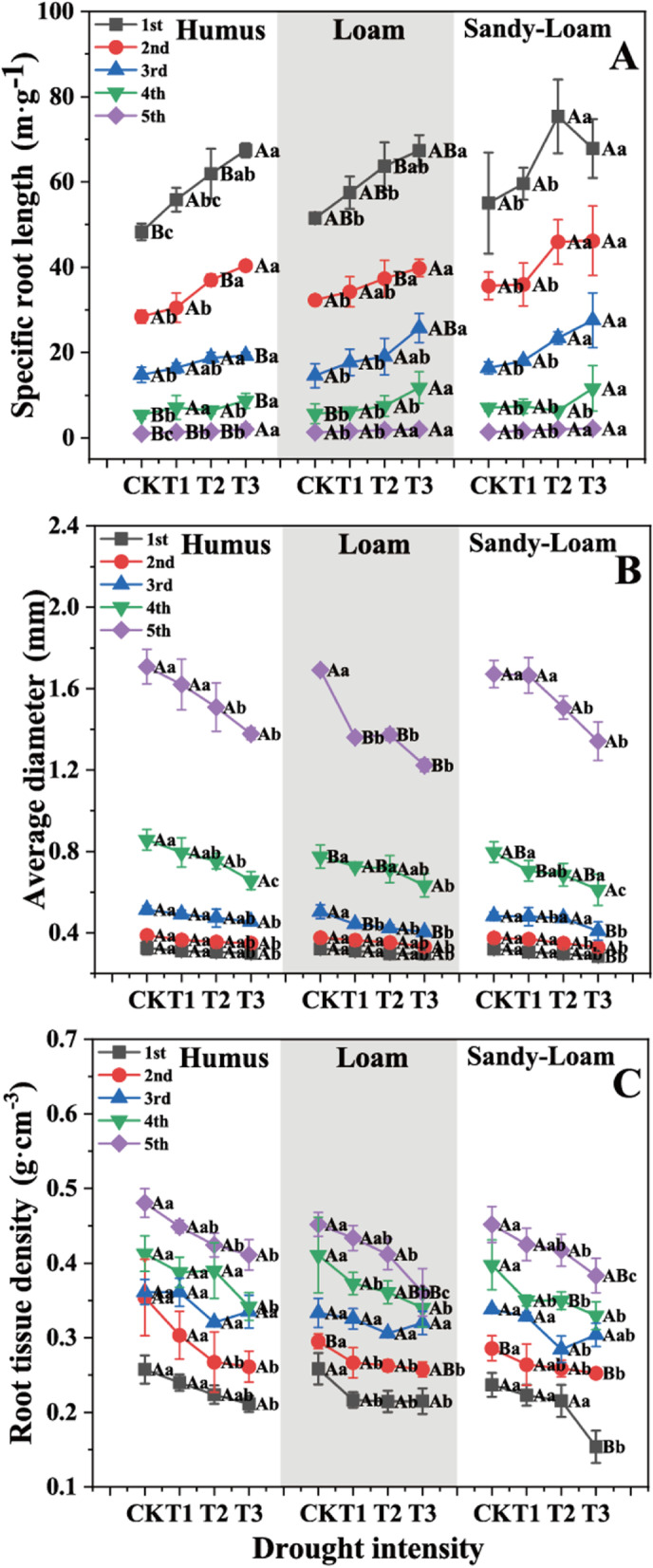
The specific root length (**A)** average diameter (**B)** root tissue density (**C)** of *J. mandshurica* seedlings in different drought intensities and soil substrates. Different lowercase letters indicate significant differences among drought intensities (*P* < 0.05). Different uppercase letters indicate significant differences among soil substrates (*P* < 0.05). *CK* control, *T1* mild drought, *T2* moderate drought, *T3* severe drought

Drought intensity had a significant effect on the SRL, AD and RTD among root branch orders (*P* < 0.05, Table S1). In general, with an increase in drought intensity, the SRL, AD and RTD of the first five order roots increased, decreased, and decreased, respectively (Fig. 2A-C). Compared with CK, the SRL of the first five order roots in T3 increased by 31.2, 31.6, 58.2, 78.5 and 70.7% (average for soil substrates) (Fig. 2A), and the RTD of the first five order roots in T3 decreased by 23.2, 16.9, 7.2, 17.2 and 16.6% (average for soil substrates) (Fig. 2C), respectively. The seedlings in sandy-loam soil had the highest SRL, and lowest AD and RTD under the same drought intensity. Compared with humus soil, the SRL of the first five order roots in sandy-loam soil increased by 10.%, 20.5, 22.3, 16.8 and 25.5% (average for drought intensities) (Fig. 2A), respectively. Soil substrate had no effect on AD among root orders.

### Effect of drought and soil substrate on fine root anatomical traits

**Fig. 3:**
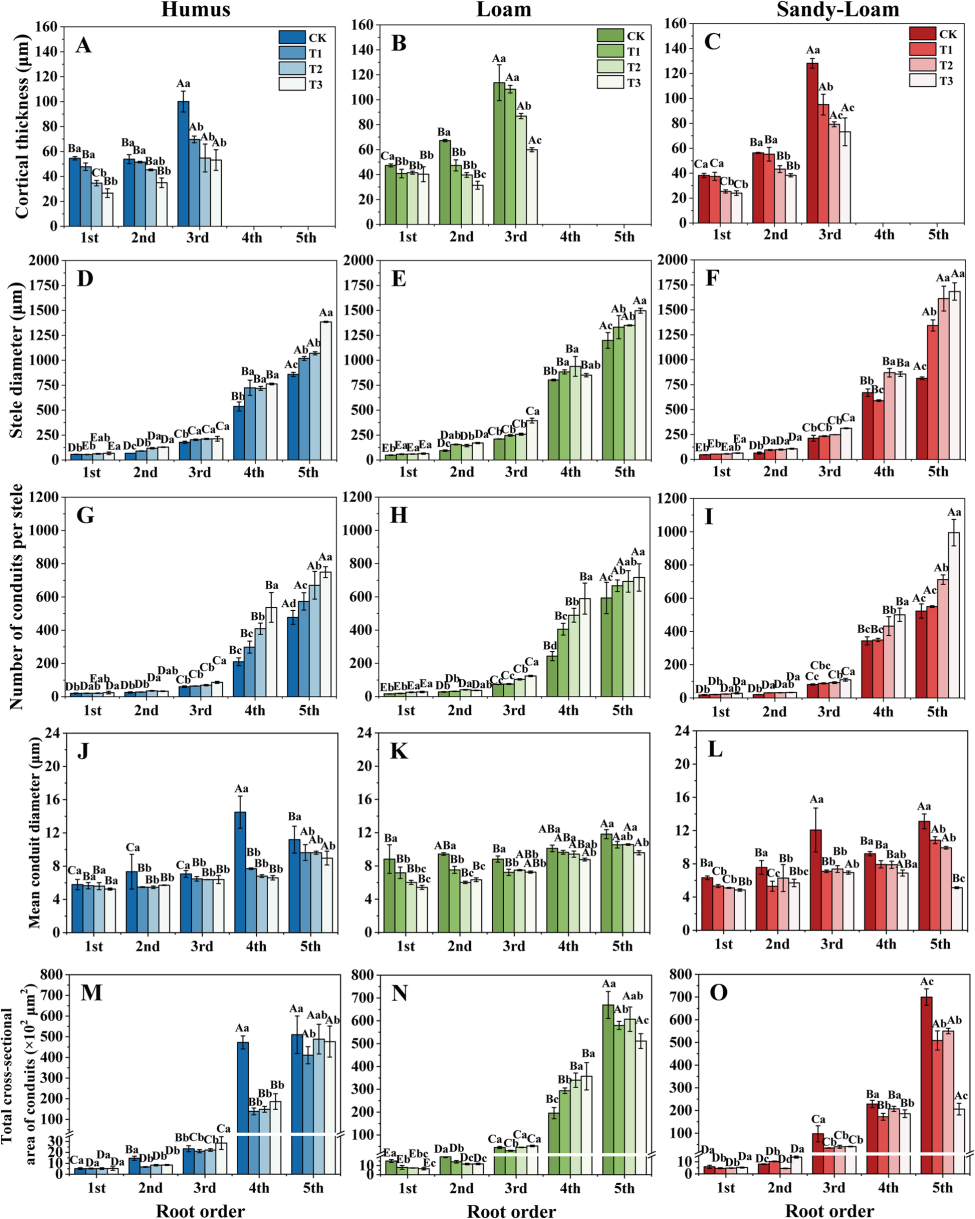
The cortical thickness (**A-C**), stele diameter (**D-F**), number of conduits per stele (**G-I**), mean conduit diameter (**J-L**) and total cross-sectional area (**M-O**) of *J. mandshurica* seedlings in different drought intensities and soil substrates. A, D, G), humus soil; B, E, H), loam soil; C, F, I), sandy-loam soil. Different lowercase letters indicate significant differences among drought intensities (*P* < 0.05). Different uppercase letters indicate significant differences among root orders (*P* < 0.05). *CK* control, *T1* mild drought, *T2* moderate drought, *T3* severe drought

Drought intensity and soil substrate had a significant effect on the CT, SD, and NCPS among root branch orders (*P* < 0.05, Table S1). With an increase in drought intensity, the CT of seedlings in the three soil substrates was reduced (Fig. 3A-C). Conversely, the SD and NCPS increased with increasing drought intensity (Fig. 3D-3I). The SD of the first five order roots in T3 were 28.6, 79.6, 51.5, 25.3 and 64.4% higher than those in CK, respectively (average for soil substrates) (Fig. 3D ~ 3 F). The NCPS of seedlings in sandy-loam soil was higher than that in humus and loam soil, and with an increase in drought intensity, the NCSP in T3 had the largest increase (compared with CK), with significant increases of 61.6, 66.0 and 58.2%, respectively (average for root orders) (Fig. 3G-3I). Soil substrate had a significant effect on MECD (*P* < 0.05, Table S1, Fig. 3J-L). Drought and root order had a significant effect on TCAC (*P* < 0.05, Table S1, Fig. 3M-O).

### Effect of drought and soil substrate on fine root NSCs content

**Fig. 4:**
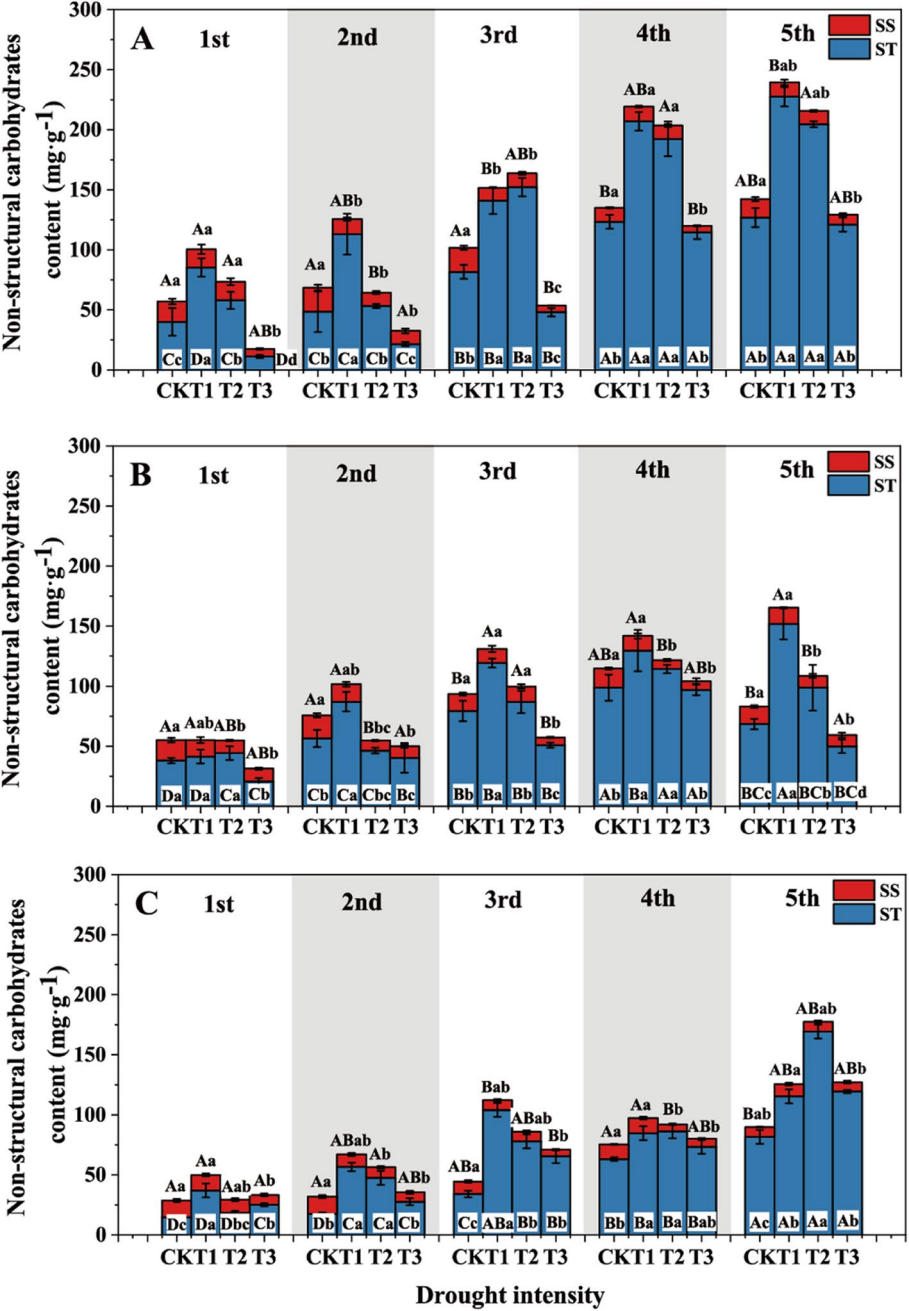
The fine root NSCs content of *J. mandshurica* seedlings in different drought intensities and soil substrates. The histogram represents the soluble sugar and starch content. Different lowercase letters denote significant differences among drought intensities (*P* < 0.05). Different uppercase letters denote significant differences among root orders (*P* < 0.05). **A**, Humus soil; **B**, Loam soil; **C**, Sandy-Loam soil. *CK* control, *T1* mild drought, *T2* moderate drought, *T3* severe drought

The fine root SS, ST and NSC contents responded significantly to drought intensity, soil substrate and root order (*P* < 0.05, Table S1). The ST content of fine roots increased under mild drought (T1) and decreased under severe drought (T3). Under the same drought intensity (except for T3), the SS contents of the first three order roots in humus soil were 26.8, 29.5 and 55.4% higher than those in sandy-loam soil with ascending root order (average for drought intensities) (Fig. 4A, C). The fine root SS content decreased with increasing drought intensity. With ascending root order, the fine root NSC content of the first five order roots in T3 decreased by 46.9, 47.0, 58.1, 51.0 and 29.4% compared with those in CK, respectively. Compared with the CK, the NSC content of first five order roots in T3 decreased by 32.1, 25.1, 8.8, 4.7% and increased by 1.3%, respectively. The ST contents of the first-order roots from humus soil in CK, T1 and T2 were 169.5, 130.5 and 217.0% higher than those in sandy-loam soil (*P* < 0.05). Except for second-order roots in loam soil, the fine root ST content in CK was lower than those in T1 and T2 but higher than that in T3 (Fig. 4A-C). In general, the root ST content increased with ascending root order. The interactions between drought and soil substrate, drought and root order exhibited a significant effect on the NSCs contents (Table S1).

### Redundancy analysis and variance partitioning analysis

**Fig. 5:**
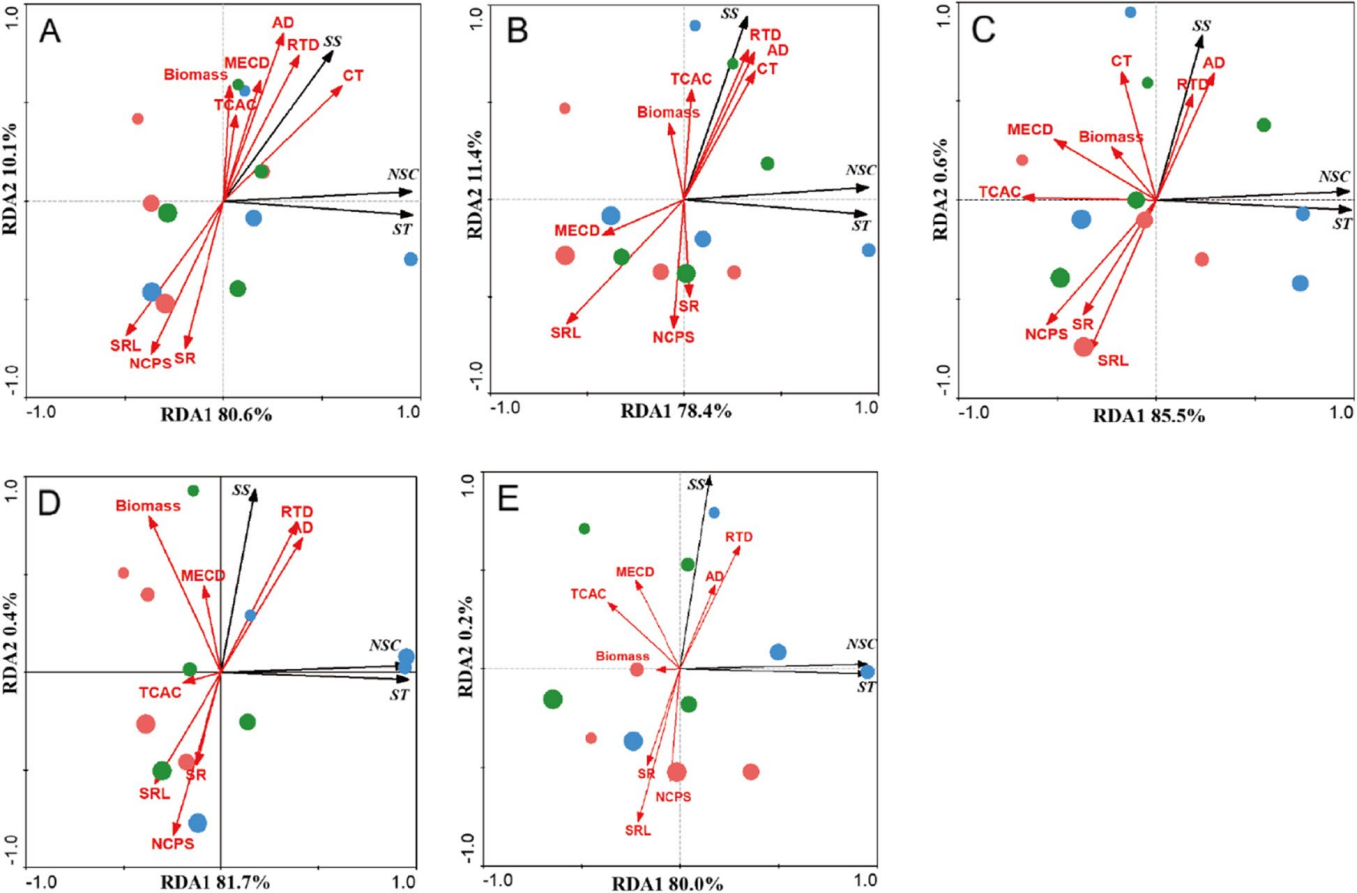
Redundancy analysis of fine root morphological and anatomical traits in different drought intensities and soil substrates. **A** 1st-order root, **B** 2nd-order root, **C** 3rd-order root, **D** 4th-order root, **E** 5th-order root. Blue symbols, Humus soil; Green symbols, Loam soil; Red symbols, Sandy-Loam soil. *SRL* specific root length, *AD* average diameter, *RTD* root tissue density, *CT* cortical thickness, *SD* stele diameter, *NCPS* number of conduits per stele, *MECD* mean conduit diameter, *TCAC* total cross-sectional area, *SS* soluble sugar, *ST* starch, *NSC* nonstructural carbohydrates

**Fig. 6:**
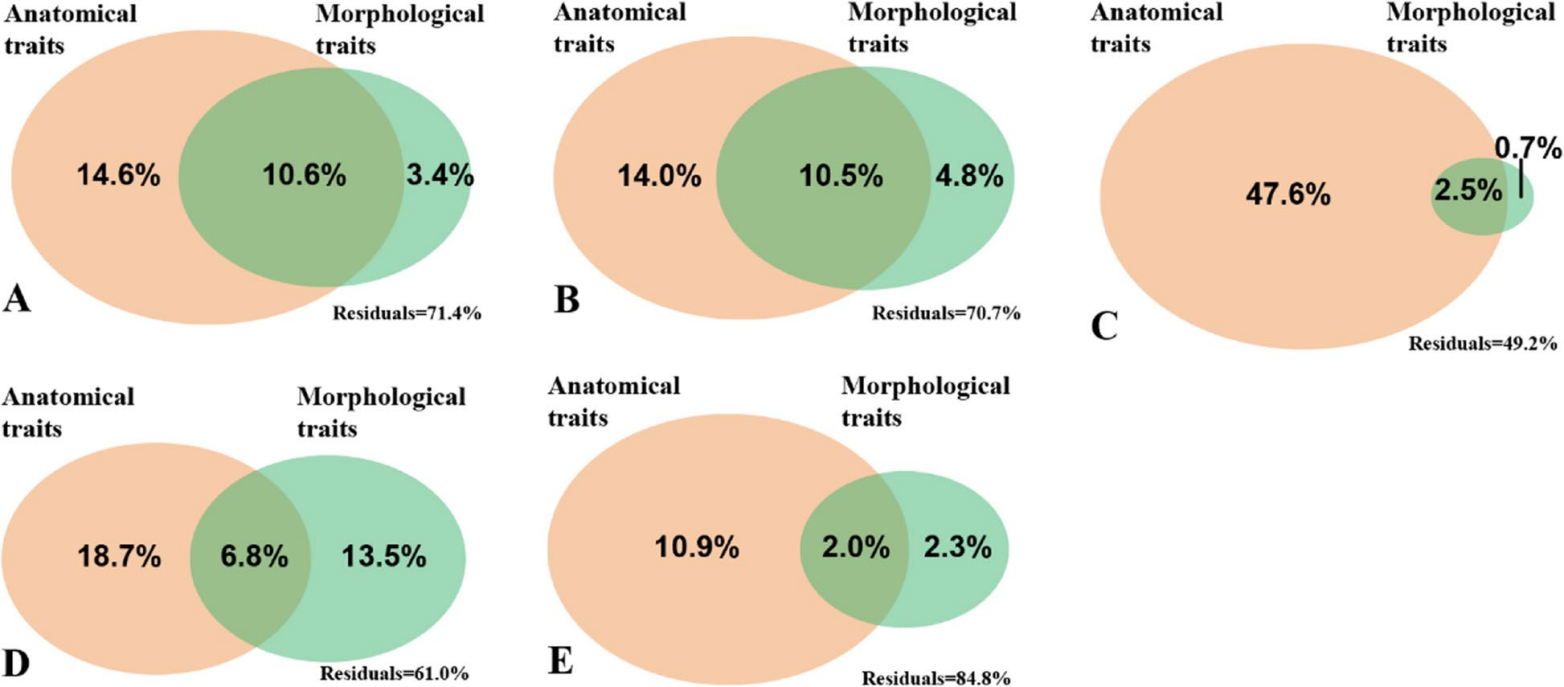
The variation partition analysis of fine root traits and root NSCs. **A** first-order root, **B** second-order root, **C** third-order root, **D** fourth-order root, **E** fifth-order root

The redundancy analysis (RDA) of the first five order roots was performed by analysing the root traits and NSCs under different drought intensities and substrates (Fig. 5A-E). The results showed that the RDA 1 and RDA 2 axes represented the variation in ST (or NSC) and SS, and the degrees of separation of drought were better than that of the soil substrate. The results of the Mantel test showed that SRL, RTD, CT, SD, NCPS and TCAC could explain the variation in root NSC contents, while AD had a smaller explanation for root NSCs (Table 1). The contribution of morphological and anatomical traits to the variation in fine root NSCs was quantitatively assessed using variation partition analysis (VPA). The explained variation in anatomical traits for the first five order roots was higher than that in morphological traits, and the NSC variation in the absorption roots (first two order roots) jointly explained by them was higher than that in transport roots (Fig. 6A-E).

**Table 1:** Mantel test on the relationship between the root traits and NSC variables

Root traits	1st-order		2nd-order		3rd-order		4th-order		5th-order	
	*R*^*2*^	*P*	*R*^*2*^	*P*	*R*^*2*^	*P*	*R*^*2*^	*P*	*R*^*2*^	*P*
SRL	0.193	**0.033**	0.346	**0.002**	0.193	**0.027**	0.099	0.168	0.103	0.174
AD	0.085	0.239	0.156	0.063	0.128	0.105	0.097	0.163	0.015	0.764
RTD	0.235	**0.018**	0.380	**0.002**	0.095	0.200	0.252	**0.007**	0.146	0.064
Biomass	0.034	0.539	0.012	0.796	0.041	0.497	0.282	**0.005**	0.002	0.957
CT	0.342	**< 0.001**	0.392	**0.001**	0.255	**0.008**	——	——	——	——
SD	0.104	0.172	0.182	**0.038**	0.285	**0.006**	0.177	**0.043**	0.131	0.094
NCPS	0.186	**0.031**	0.267	**0.006**	0.383	**0.002**	0.372	**< 0.001**	0.174	**0.033**
MECD	0.010	0.836	0.136	0.090	0.209	**0.014**	0.083	0.252	0.107	0.127
TCAC	0.010	0.826	0.229	**0.023**	0.261	**0.007**	0.006	0.924	0.044	0.476

*SRL* specific root length, *AD* average diameter, *RTD* root tissue density, *CT* cortical thickness, *SD* stele diameter, *NCPS* number of conduits per stele, *MECD* mean conduit diameter, *TCAC* total cross-sectional area. Significant correlation coefficients (*P* < 0.05) are shown in bold

## Discussion

Our work revealed several key findings related to the variation in Manchurian walnut seedling root traits and NSCs induced by different soil substrates and drought intensities. The fine roots in humus soil had a higher NSC (SS and ST) content. The root SS contents exhibited a decreasing trend with an increase in drought, while the root ST contents showed an increase and a decrease under mild drought and severe drought, respectively. Interestingly, the anatomical traits explained the higher variation in the NSC content (the first five order roots). Compared with the transport roots, the morphological and anatomical traits jointly affected the higher variation in NSC content of the absorptive roots (Fig. 6). These results imply that the physiological response of the absorptive roots may be more sensitive to the surrounding environment than transport roots.

### Root morphological and anatomical traits vary among different drought intensities and soil substrates

**Fig. 7:**
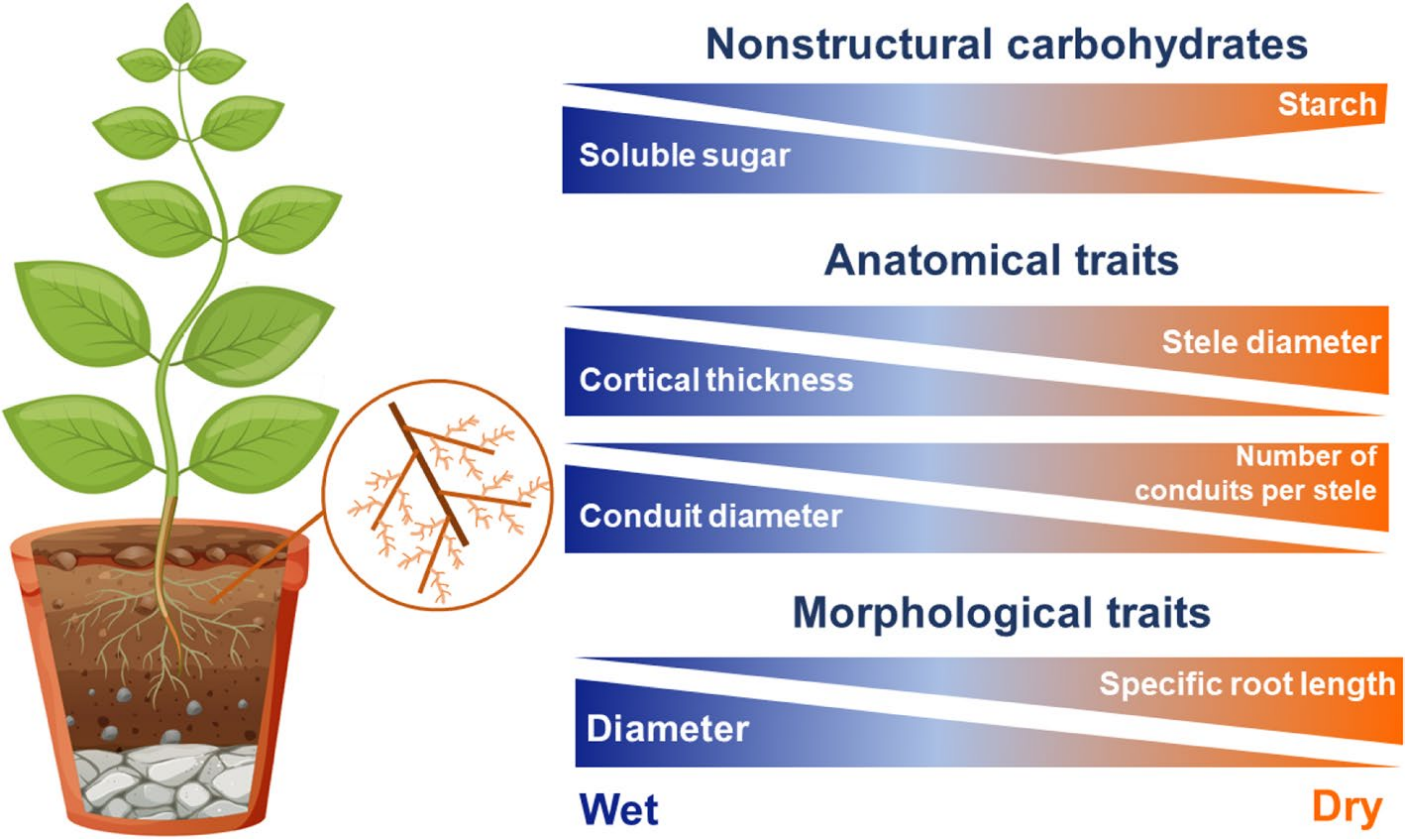
Conceptual paradigm synthesizing the patterns from this study into observed associations between fine root traits and nonstructural carbohydrates across the progressive drought treatments

Root traits have long been proposed as a major avenue of research to determine how plants respond to water limitations and their adaptation to acquire resources [4, 6, 39]. In this study, drought decreased the AD, RTD, and CT of Manchurian walnut seedlings, and increased the SRL and SD (Fig. 7). These drought-driven plastic responses of fine roots supported our first hypothesis; that is, root morphological and anatomical traits coordinately varied to maintain the plant survival under water constraints. Plants can adapt to water deficit through adjustments in their structure to facilitate water absorption and transport capacity [40]. In this study, the seedlings in sandy-loam soil had a greater SRL than those in humus soil, which is due to the characteristics of sandy loam soils with lower porosity, larger water holding capacity, and lower N content under the same drought conditions (Table 2; Fig. 2A). Based on the dataset of 1115 species on the global scale, Freschet et al. [41] found that soil bulk density strongly affected root morphological traits via adaptation for environmental variation. Lynch et al. [13] revealed that allocating resources to elongation growth insteaded of radial thickening contributes to improve efficiency in soil exploration. Additionally, Ma et al. [6] indicated that even minor changes in RTD could have a significant impact on the volume of soil explored per unit of carbon investment. In this work, our results showed that drought significantly reduced the RTD of the first five order roots, while the RTD of Manchurian walnut seedlings in sandy loam soil was significantly lower than that in humus soil (Fig. 2C). Fine roots with low RTD have higher metabolic efficiency, lower hydraulic conductivity, root lifespan, and ability to penetrate solid soil in the process of soil resource capture [33, 42].

**Table 2:** The initial growth of seedlings and physicochemical properties of three soil substrates before drought experiment

Seedlings/Soil substrate property	Humus soil	Loam soil	Sandy-Loam soil
Bulk density (g·cm^− 3^)	1.18 ± 0.02b	1.35 ± 0.02a	1.32 ± 0.02a
Total porosity (%)	53.19 ± 1.12b	58.90 ± 0.91a	36.72 ± 0.95c
Aeration porosity (%)	25.61 ± 0.48a	23.52 ± 0.53a	13.01 ± 0.99b
Water absorption capacity	0.23 ± 0.01b	0.26 ± 0.01a	0.18 ± 0.01c
Penetrate rate (g·min^− 1^)	4.49 ± 0.04a	4.01 ± 0.12b	3.04 ± 0.18c
Evaporation rate (g·h^− 1^)	0.59 ± 0.01a	0.50 ± 0.01b	0.37 ± 0.01c
Total nitrogen (mg·g^− 1^)	7.09 ± 0.78a	3.11 ± 0.05b	1.20 ± 0.03c
Total phosphorus (mg·g^− 1^)	0.71 ± 0.04a	0.34 ± 0.09b	0.32 ± 0.01b
Available phosphorus (mg·kg^− 1^)	13.10 ± 0.82a	5.04 ± 0.21b	12.75 ± 0.69a
Height (cm)	58.9 ± 3.70a	55.0 ± 4.62a	50.6 ± 4.77a
Basal diameter (mm)	7.68 ± 0.90a	7.14 ± 0.62a	6.77 ± 0.43a

Different letters in the same line indicate significant differences among the different treatments (*P <* 0.05)

The number, diameter and area of xylem conduits affect axial water transport, while cortical traits and lignified cell layers may affect radial water transport [43]. Rieger and Litvin [44] found that the thinner the diameter (or the cortex thickness), the greater the water absorption capacity, because water absorbed by fine roots must traverse the cell layers in the radial pathway before reaching the stele [45]. In this study, drought significantly decreased and increased the fine root CT and SD of Manchurian walnut seedlings, respectively, which implied that Manchurian walnut seedlings modified a thinner cortex under water deficit, to reduce the metabolic cost of root elongation and explore more soil resources. Additionally, when exposed to drought, the Manchurian walnut seedlings will strategically adjust the conduit diameter and conduit density, and a smaller conduit diameter will be beneficial for generating greater water tension. More importantly, fine root NCPS was higher in hostile environments (sand-loam or severe drought), which indicated that plants exhibit a survival strategy to enhance hydraulic conductivity and water transport efficiency [46]. Additionally, Chimungu et al. [47] demonstrated that roots with smaller cells in the cortex were less vulnerable to buckle and collapse during penetration at high bulk density. A lower MECD was observed in sandy-loam soil, especially in the severe drought regime. Guo et al. [9] found that variations in fine root functions for transportation and absorption were associated with anatomical traits based on branching orders. The diameter and cortex thickness were more pronounced in higher-order roots under severe drought, and the coefficients of variation in SRL and NCPS in higher-order roots subjected to drought were higher than those in lower order roots, because branch orders play different roles in ecosystem carbon and nutrient cycling [48], further, lower-order roots have higher turnover and respiration rates [49, 50]. Therefore, based on the perspective of branch orders, we should pay more attention to the water absorption traits of the lower-order roots and the water transport traits of the higher-order roots, which are equally important under adversity. Taken together, our findings suggest that root morphological and anatomical traits of Manchurian walnut seedlings coordinately vary to adapt the water deficit and change of soil substrates.

### Drought- and soil substrate- induced variation in root NSCs

The reserve of NSCs among plant organs is important for plants to survive in harsh environments, and they reflect the trade-off between photosynthesis and respiration, which may affect carbon acquisition for growth depending on the species-specific living strategy [22, 51]. Based on the meta-analysis of 52 tree species worldwide, He et al. [52] demonstrated that variations in NSCs of plants were associated with drought intensity, the net loss of carbohydrates in roots was significant, and the plant NSCs decreased only under severe drought. In this study, compared with severe drought, higher root NSC contents under mild drought (T1 treatment) provided a survival advantage for seedlings (Fig. 7), which was consistent with the study of O’Brien et al. [51]. We found that the SS and ST contents of fine roots (first five-order roots) of Manchurian walnut seedlings in the three substrates decreased and increased, respectively, which is consistent with the study of Hagedorn et al. [53], who found that the root NSCs of seedlings under drought will accumulate (mainly increasing the starch content). Indeed, trees may prioritize investment in belowground assimilation to restore root function after drought [53]. Many studies have shown that the NSC contents of plants increase in the early stage of drought or under mild stress [54–56]; however, when water deficit becomes more unfavourable (under long-term drought or severe drought), trees may suffer from a striking reduction in NSCs in the whole plant or specific organs [22, 25, 51]. Gaul et al. [57] concluded that the production of fine roots was stimulated under mild drought, which indicates that plants compensate for the increase in root mortality caused by the requirement for water. On the other hand, plants exposed to severe drought shed old fine roots, and do not produce new or more active fine roots. When the pathway of NSCs to the fine roots is blocked, the carbohydrates produced by photosynthesis are not transported belowground, and then fine roots only show a preference for regulating their own NSC levels to cope with drought stress [22]. However, this inference needs to be verified in combination with the temporal dynamics of fine root NSCs in further studies.

More notably, the NSC contents of fine roots in the humus soil were higher than those in the sandy-loam soil. As more nitrogen is absorbed by the fine roots in humus soil, the corresponding carbohydrates must be invested to maintain metabolism [58]. Previous studies have found that seedlings of the drought-tolerant species *Pinus tabuliformis* invest more carbon in lower-order roots to facilitate plants to absorb more water and nutrients to survive under harsh conditions [27]. Similar results were also presented in our recent study on *Fraxinus mandshurica* [30], after 60 days of drought, the lower-order roots of *F. mandshurica* seedlings had higher NSC contents than those of higher-order roots. This result implies that when carbon is limited, *F. mandshurica* seedlings preferentially allocate carbon to thinner roots rather than thicker roots. In this study, our results were different from those of the two studies above, the lower–order roots of Manchurian walnut seedlings had a higher SS content and a lower ST content. With ascending root order, the root NSC content increased, which supported the third hypothesis. Compared with *P. tabuliformis* and *F. mandshurica*, Manchurian walnut seedlings have lower drought tolerance (naturally distributed in well-drained hillsides or moist loose soils with more humus on riverbanks), and they may store more carbon in higher order roots to reduce the cost caused by the high turnover rate of lower-order roots. Jia et al. [59] revealed that the NSC to N ratio increased from lower order roots to higher order roots, which indicated that NSCs assigned to first-order roots will be consumed faster than those assigned to fifth-order roots due to the higher respiration of root tips. Collectively, our results emphasize that the NSC content of higher order roots in Manchurian walnut seedlings varied more resilient to drought compared with lower order roots.

### Root traits had a strong association with the variations of root NSCs to drought and soil substrate

Mounting evidence has suggested that thick roots increase NSC accumulation [60, 61], whether root traits are associated with NSC contents remains to be tested. Drought can directly increase root mortality by depleting starch and sugar reserves, and indirectly inhibit the transport of photosynthate to the root system [62]. Yang et al. [28] found that the root NSC content of *Phyllostachys edulis* seedlings under drought was closely related to SRL and root architecture. However, the relationship between the fine root traits and hierarchy allocation of NSC contents among branch orders remains to be verified. In this study, the SRL and RTD of the first two root orders exhibited a strong association with the variation in fine root NSC content. In contrast to our previous studies [29, 30], fine root diameter did not significantly explain the variation in the higher content of fine root NSCs. Wang et al. [63] showed that the average root elongation rate was significantly negatively correlated with soluble sugar and positively correlated with starch content.

Root anatomical traits are strongly related the variation in root traits and the balance between plant resource acquisition and investment [6, 64]. Yamauchi et al. [16] showed in a recent study that the ratio of root tissue area (cortex to stele ratio, xylem–to–stele ratio and aerenchyma–to–cortex ratio) were three key anatomical traits that could explain the adaptability of wild Poaceae plants to the soil moisture gradient. Generally, axial water conductance is influenced by the xylem vessel traits (number, diameter and area), while radial conductance may be impacted by cortical traits and the presence of suberized cell layers [65]. The smaller vessels may contribute to the regulation of water flow under water-limiting growth conditions and may be less vulnerable to xylem embolism caused by drought [4]. In this study, the root anatomical traits from different branch orders (especially CT, NCPS and SD) explained a higher proportion of the variation in fine root NSCs content (compared with morphological traits). For lower-order roots, both the morphological and anatomical traits explained the high proportion (for first-order root and second-order roots, 10.6% and 10.5%, respectively) of the variation in fine-root NSCs induced by drought and soil substrate, which supports our fourth hypothesis. Under adverse conditions, plants reduce the metabolic cost of soil resource capture by reducing nutrient content and respiration, and promote greater soil exploration [13]. Due to the respiration of cortical cells consuming water and nutrients, the allometric growth of branch order roots determines the trade-off between carbon supply and consumption [66]. Lynch et al. [13] revealed that the formation of root cortical aerenchyma under drought would reduce the metabolic cost of soil exploration, as well as the nutrient content and respiration by transporting living cortical parenchyma to space. Meanwhile, the reduction in cortex thickness is also conducive to the radial transport of water from the root system to the vascular tissue. However, our research only focused on the impact of drought on fine roots in a short-term manipulation experiment, and we did not measure the hydraulic conductivity of fine roots. Given the low carbon input of photosynthate to roots during long-term drought, it is necessary to apply isotope labelling technology combined with long-term drought experiments to clarify a more comprehensive mechanism in future research.

## Conclusion

This study provides unique observations and valuable information for the first time about the coordinated relationship between fine root traits and NSC content under different drought intensities and soil substrates. Our results indicated that Manchurian walnut seedlings synergistically adapted their fine root morphological and anatomical traits under different drought and soil substrates. In addition, the fine root SS content decreased with increasing drought intensity, and the ST content of fine roots under mild drought increased and decreased under severe drought. The variation in NSCs in lower-order roots showed a strong association with morphological and anatomical traits, while the NSC variation in higher-order roots was mainly explained by anatomical traits (cortical thickness and number of conduits per stele). From the viewpoint of root hierarchic structure (branch root orders), our results highlight the coordinated adaptation of the root traits and NSCs of Manchurian walnut seedlings exposed to water gradients in different soil substrates. We propose that the potential relationship of root traits and NSC content is likely to provide important insight into the root economics spectrum.

## Materials and methods

### Experimental site and sapling preparation

The pot experiment was conducted in Xinli Town, Jingyue Development District, Changchun, China (43°33′ N-44°41′ N, 125°19′ E-125°24′ E). This site has a temperate continental monsoon climate, with a frost-free period of 140 days, a mean annual rainfall of 600 ~ 800 mm and a mean annual temperature of 4.6 ℃. The seeds of *J. mandshurica* were collected by the Hongwei nursery in the Lushuihe Forestry Bureau in September 2015. A voucher specimen of this material has not been deposited in a publicly available herbarium. In 2016, the seeds with full grains and no pests were selected for disinfection, and then cleaned with distilled water. After seed stratification, the seeds with consistent white radicles exposure were selected for sowing in the Hongwei nursery of the Lushuihe Forestry Bureau. We kept well-watered status 80–85% of the maximum field water-holding capacity) prior to the application of drought treatments. The application of fertilizer is 400 mg N-P-K compound fertilizer (N:P_2_O_5_:K_2_O = 15:15:15).

Seedlings of *J. mandshurica* (*n* = 360) with the same basal diameter and height, and relatively complete root system were selected and transplanted into plastic pots (height × diameter, 30 cm × 24 cm; volume is 0.014 L) in April 2017 (leaves emerged). The soil in the pots was taken from the nursery, and the humus soil was collected in the secondary forests in the Lushuihe Forestry Bureau. The soil type is a Eum-Orthic Anthrosol according to the Food and Agricultural Organization soil classification system. Humus, loam and sandy-loam (sandy and loam, 1:1, w/w) soils were filled into pots with equal volume. The pots were placed in rows 0.5 m apart from the neighbours in the open and flat area under the rainout shelter. The soil moisture was maintained in a suitable status (80% of field water-holding capacity) to ensure that seedlings grew rapidly to survive the rejuvenation period.

### Experimental design and sampling

In July 2017, soil samples from the three soil substrates were measured before the beginning of the drought manipulation. Uniform *J*. *mandshurica* seedlings (for each treatment, n = 30) were randomly selected from three different soil substrates for drought experiments (Table 2) [29, 30]. A two-factors complete orthogonal design (three soil substrates and four drought intensities) was conducted with the soil substrates and drought. The drought experiment was set to four drought intensities, CK (80 ~ 85% of the maximum field water-holding capacity, FC), mild drought (T1, 60%~65% FC), moderate drought (T2, 40 ~ 55% FC), and severe drought (T3, 20 ~ 25% FC). Briefly, the soil moisture content of the pots was monitored by weight. The pots were weighed before the progressive drought experiment, and then the theoretical weight was calculated for different drought intensities of the three substrates. These pots were thereafter controlled daily to maintain a constant weight until the experiment ended in September 2017. A detailed description of the drought control is provided in our previous study [29, 30].

After two months of continuous drought stress (from July to September 2017), the roots of *J. mandshurica* seedlings were collected. Seedlings (n = 10) were randomly selected in each experimental block. The root was sorted carefully and separated from the soil, and the soil particles were washed off the root samples with deionized water until the branch structure of the roots could be identified. The root samples were separated into two subsamples, and one set of subsamples was immediately fixed in formalin-aceto-alcohol solution (FAA, 90 ml of 70% ethanol, 5 ml 100% glacial acetic acid and 5 ml glycerol) for anatomical measurement, and refrigerated (2 ~ 3 ℃) for morphological and anatomical analysis of the roots. In this study, we only measured live root samples, and dead roots were picked and discarded.

### Soil physicochemical property and fine root NSCs concentration

In June 2017, soil samples from three substrates were collected, and the soil physicochemical properties were measured (Table 2). The total soil nitrogen was determined with Kjeldahl digestion (Tecator Kjeltec Auto 1030, FOSS TECATOR, Swedish). The soil total phosphorus and available phosphorus were measured using the colorimetric method and double acid extraction, respectively (Yang et al., 2018). The soil bulk density, aeration porosity, total porosity, water absorption capacity, penetration rate and evaporation rate were measured by referring to the description by Ji et al. [29, 30].

NSCs consisted of soluble sugar (SS) and starch (ST), and the sum of their concentrations was determined by the anthrone method [67]. Briefly, a 10 ml centrifuge tube containing root samples (0.1 g) was filled with 2 ml of 80% ethanol. After being incubated for 30 min at 80 °C in a water bath. The mixture was centrifuged for 5 min at 4,000 rpm. The supernatant was transferred in a new centrifuge tube, and the sugars were re-suspended and extracted from pellets two more times using 80% ethanol. The supernatant was retained, combined, and stored at − 20^◦^C for SS determination. Subsequently, all collected supernatants were pooled and dried at 55 °C until all of the solventd had been evaporated, and starch was then extracted from the ethanol-insoluble pellet. The ST in the residue was then released by boiling in 2 ml distilled water for 15 min. The pellet was further suspended in 2 ml of 9.2 M HClO_4_ and incubated at 80 °C for 30 min. About 4 ml of distilled water was then added, and the mixture was centrifuged at 4,000 rpm for 5 min. A further extraction was carried out with 2 ml 4.6 M HClO_4_. The supernatant was also retained, combined, and stored at − 20^◦^C for ST determination. The NSC contents were determined by ultraviolet spectrophotometry (New Century 6T, Beijing Purkinje General Instrument Co. LTD., Beijing) [67]. The sugar concentration was calculated by the regression equations based on glucose standard solutions and the starch concentration, with the starch content multiplied by a conversion factor of 0.9 [68].

### Fine root morphological and anatomical traits

In the laboratory, root samples were placed in a petri dish containing deionized water and divided into different root orders. The classification was conducted according to the root order classification method of Pregitzer et al. [69]. The root samples were scanned with a scanner with a resolution of 500 dpi (Expression 10000XL, Epson Telford Ltd, Telford, UK). The average diameter, total root length and volume were measured with the root system analyser software WinRhizo (2004b, Regent Instruments, Inc., Québec, Canada). The scanned roots were oven-dried to constant weight at 65 ℃ to measure the root dry mass. Finally, the specific root length (SRL), specific root surface area (SRA) and root tissue density (RTD) were calculated according to our previous description of Ji et al. [29, 30].

The root subsamples in FAA solution were removed and the anatomical analysis samples were dissected based on branch order [69]. A total of 10 ~ 20 root subsamples of the first five order roots in each treatment were randomly selected, and the cross-section of fine roots was made by paraffin section technology. A detailed description can be found in Guo et al. [9]. The slides were observed using OLYMPUS BX-51 biological microscope (Olympus Electronics Ins., Tsukuba, Japan). The cortical thickness (CT), stele diameter (SD), number of conduits per stele (NCPS), mean conduit diameter (MECD), and total cross-sectional area of conduits (TCAC) of the first five order roots were measured and calculated with Motic Images Advanced 3.2 software (Motic Corporation, Zhejiang, China).

### Data analysis

The fine root traits and NSC content were tested for normality and variance homogeneity requirements, and then three-way ANOVA was performed with drought intensity, soil substrate and root order as fixed factors to identify the significant effects by SPSS 19.0 (IBM Co., Armonk, NY, USA). The differences among treatments were assessed using Tukey’s honest significant difference test. A redundancy analysis (RDA) was performed to detect all branch orders of the association between the root traits (morphological and anatomical traits) and NSC content (using the mean values for each group) under different treatments by Canoco software (Version 4.56, Biometrics Plant Research International Wageningen, The Netherlands). R (Version 4.0.5) software (vegan package) was used to perform the Monte Carlo test for the root traits (R Core Team, 2013). Using the “vegan” package in R software (Version 4.0.5), variation partitioning analysis (VPA) was conducted to explore the individual and joint effects of morphological and anatomical traits on explaining the variations in NSCs. All data are the mean ± standard error (mean ± SE). All bar figures were drawn using Origin Pro 8.5 (OriginLab, Northampton, MA, USA).

## Supplementary Information

**Additional file 1.** 

## Data Availability

The datasets used and/or analysed during the current study are available from the corresponding author on reasonable request.
