# Supplementary material for: Drought- and soil substrate-induced variations in root nonstructural carbohydrates result from fine root morphological and anatomical traits of Juglans mandshurica seedlings
Source: BMC Plant Biol. 2023 Feb 7;23:83. doi: 10.1186/s12870-022-03987-x (PMC9903586; doi:10.1186/s12870-022-03987-x)
Supplement: Supplementary file 1 — Additional file 1. [file 12870_2022_3987_MOESM1_ESM.docx]

Supplementary materials

Original research

Title: Drought- and soil substrate-induced variations in root nonstructural carbohydrates result from fine root morphological and anatomical traits of *Juglans mandshurica* seedlings

**Li Ji^1,2,3^, Jun Wang^2^, Yue Liu^2^, Zhimin Lu^2^, Witoon Purahong^3*^, Yuchun Yang^2*^**

^1^ School of Forestry, Central South University of Forestry and Technology, 410004 Changsha, P.R. China

^2^ Jilin Academy of Forestry, Changchun 130033, P.R. China

^3^ UFZ-Helmholtz Centre for Environmental Research, Department of Soil Ecology, Theodor-Lieser-Str. 4, Halle (Saale) 06120, Germany

*** Correspondence:**

Witoon Purahong, E-mail: witoon.purahong@ufz.de

Yuchun Yang (Correspondence), E-mail: [yang-yu-chun@163.com](mailto:yang-yu-chun@163.com); 3528 Linhe Street, Changchun 130033, P.R. China; Phone number: 0431-85850434

**Running title:** Coordinated variation in root NSCs and traits

**Table S1** Results of three-way (drought intensity × soil substrate × root order) ANOVA of fine roots biomass, traits and NSCs.

| Parameter | Drought | Soil substrate | Root order | D×S | D×R | S×R | D×S×R |
| --- | --- | --- | --- | --- | --- | --- | --- |
| SRL (m·g^-1^) | **<0.001** | **0.004** | **<0.001** | 0.932 | **0.015** | 0.635 | 1.000 |
| AD (mm) | **0.005** | 0.348 | **<0.001** | 0.996 | 0.493 | 0.896 | 1.000 |
| RTD (g·cm^-3^) | **<0.001** | **0.014** | **<0.001** | 0.998 | 0.971 | 0.997 | 1.000 |
| Biomass (g) | **0.001** | **0.012** | **<0.001** | 0.758 | 0.972 | 0.880 | 1.000 |
| CT (μm) | **<0.001** | **0.001** | **<0.001** | 0.893 | **<0.001** | **<0.001** | **0.011** |
| SD (μm) | **<0.001** | **<0.001** | **<0.001** | **<0.001** | **<0.001** | **<0.001** | **<0.001** |
| NCPS | **<0.001** | **<0.001** | **<0.001** | **0.009** | **<0.001** | **<0.001** | 0.180 |
| MECD (μm) | 0.281 | **<0.001** | **<0.001** | 0.299 | 0.295 | **<0.001** | 0.284 |
| TCAC (μm^2^) | **0.005** | **0.001** | **<0.001** | 0.213 | 0.070 | **<0.001** | **0.005** |
| SS (mg·g^-1^) | **<0.001** | **<0.001** | **<0.001** | **0.003** | **0.005** | 0.566 | 0.257 |
| ST (mg·g-^1^) | **<0.001** | **<0.001** | **<0.001** | **<0.001** | **<0.001** | **<0.001** | **0.005** |
| NSC (mg·g^-1^) | **<0.001** | **<0.001** | **<0.001** | **<0.001** | **<0.001** | **<0.001** | **0.007** |

SRL, specific root length; AD, average diameter; RTD, root tissue density; CT, cortical thickness; SD, stele diameter; NCPS, number of conduits per stele; MECD, mean conduit diameter; TCAC, total cross-sectional area. D, drought. S, soil substrate. R, root order. Significant correlation coefficients (*P* < 0.05) are shown in bold.
